# Too much equity – is there such a thing? The public discourse surrounding elective egg freezing subsidy in Israel

**DOI:** 10.1186/s12939-023-01831-8

**Published:** 2023-02-18

**Authors:** Daphna Birenbaum-Carmeli

**Affiliations:** Department of Nursing, Faculty of Social Welfare and Health Sciences, University of Haifa, Haifa, Israel

## Abstract

**Background:**

The preservation of human ova for future fertilization has been made available to healthy women in 2011–2012. This treatment, dubbed elective egg freezing (EEF), is undertaken primarily by highly educated unpartnered women without children, concerned of age-related fertility decline. In Israel, treatment is available to women aged 30–41. However, unlike many other fertility treatments, EEF is not state subsidized. The public discourse of EEF funding in Israel is the focus of the present study.

**Method:**

The article analyzes three sources of data: press presentations of EEF; a Parliamentary Committee discussion dedicated to EEF funding; interviews with 36 Israeli women who have undertaken EEF.

**Results:**

Numerous speakers raised the issue of equity, claiming that reproduction was a state interest and therefore, a state responsibility, including securing equitable treatment to Israeli women of all economic strata. Highlighting the generous funding of other fertility treatments, they claimed that EEF was inequitable, discriminating against poorer single women, who could not afford it. Few actors, however, rejected state funding as intervention in women’s reproductive lives and called for reconsideration of the local reproductive imperative.

**Conclusion:**

The invocation of equity by Israeli users of EEF, clinicians and some policy makers as grounds for a call to fund a treatment that serves a well-established subpopulation seeking to relieve a social rather than a medical problem, illustrates the profound context-embeddedness of notions of health equity. More generally, it may suggest that using an inclusive language in a discourse of equity may potentially be invoked so as to promote the interests of a particular subpopulation.

Equity in health commonly looks into access and treatment of major, potentially life-threatening medical conditions, most often focusing on underprivileged populations. The present article investigates a somewhat different landscape. First, the scrutinized service is that of elective egg freezing (EEF), a fertility preservation treatment aiming to enable healthy women conceive at a later age, when motherhood is more compatible with their lives. While addressing a life-changing social and existential concern – involuntary childless – the problem at hand is social rather than medical. Second, the relevant clientele that normally undertakes the treatment is by and large pretty well-off: healthy, well-educated women in their thirties, mostly covered by comprehensive health insurance in wealthy countries. Third, ample studies show that the majority of women who undertake EEF do not return to the clinic in order to use the preserved ova. From an equity perspective, in the field of the present research, the state of Israel, public health insurance is universal and includes, among other services, the world’s most generous coverage for fertility treatments. It is against this backdrop that the lack of funding for EEF is of distinct local significance and a cause of frustration for numerous consumers, activists and some policy-makers. This article probes the public discourse surrounding EEF funding in Israel, focusing on the issue of equity, as a somewhat exceptional use of the notion, exploring its local significance alongside a potentially more general lesson to be learned on equity. The case at hand illustrates how the notion of health equity and the use of inclusive language might be invoked so as to eventually promote the interests of a particular subpopulation, which generally belongs in a fairly resource-rich segment of society.

Health policies are context-dependent, varying greatly across countries, social classes and insurers, with various bodies granting wider services in some areas than in others (e.g., [1–5]). Local ranges of service evidently reflect the composition of local problems, e.g. child malnutrition in sub-Saharan Africa [6], maternal mortality in Brazil [7] or mosquito bites in Ethiopia [8]. In affluent countries that offer citizens a wide array of treatments, going beyond basic medical needs, service composition may be even more indicative of local priorities. Thus, in England, some types of cosmetic surgeries are publicly funded [9], while Saudi Arabia is planning to invest a billion dollar a year in anti-aging research [10]. The state of Israel has been exceptional in the amount of resources it has been allocating to reproductive services, for decades.

The technology of egg vitrification enables flash freezing of a woman’s ova, so as to be thawed and fertilized later on in the woman’s life. The treatment was originally offered in the early 2000s to young cancer patients, aiming to preserve their fertility beyond chemotherapy’s damages. Since 2011–2012, treatment is also offered for non-medical reasons, primarily, on grounds of anticipated age-related fertility decline [11]. This procedure is named here elective egg freezing (EEF) [12]. Starting out like an IVF procedure, in EEF, the retrieved ova are not being fertilized and transferred to the womb but are preserved and stored in the laboratory. At present, numerous industrialized countries offer self-funded EEF services (e.g., the UK, the Netherlands, Belgium, Spain, Turkey, the U.S., Australia, Korea and Israel [13];), thereby privileging wealthier women. The only exception is France, which has ratified EEF only in 2021 and granted the service some public funding [14]. Ample studies show that the majority of women use the technology in their latter 30s, due to lack of reproductive partner [15–21]. Levels of use are relatively low. According to various studies, 3 to 38% of the women return to clinics in order to have their ova thawed and fertilized [22].

EEF is part of the expanding range of assisted reproductive technologies (ART) that are being increasingly used in the shaping of people’s lives and family ties. With the proliferation of these technologies, state regulation and issues of equity in this private domain have expanded. The impact of related policies goes beyond individuals, enhancing and potentially modifying social perceptions of key social notions such as parenthood and normativity alongside fairness and equity.

## Elective egg freezing – policy, funding, equity

Feminists are divided about EEF. Whereas some argue that the new technology expands gender and class equity, others contend it widens social gaps (e.g., [17, 23–25]). In practice, the demand for EEF is rising globally. In the UK [26, 27], Korea [28], the USA, Australia and New Zealand [29], demand for EEF has consistently expanded, as more unpartnered childless women in their thirties seek fertility preservation [12, 15–17, 19–21, 30–34]. During the Covid-19 pandemic, demand has further increased [35].

The surge in uptake may overshadow equity challenges posed by local policies. Treatment cost is a major impediment for many women. Unlike egg freezing due to medical reasons, which is often subsidized (e.g., in 14 out of 27 countries in Europe, as well as in Australia and Israel [12, 32];), EEF is privately funded [33, 36]. As mentioned, the only exception is that of France, where EEF has only been ratified in 2021, but was then granted partial subsidy, thereby challenging the established distinction between medical and non-medical EF as a main funding criterion [22].

Though scholars debate the clarity of the division between medical and non-medical reasons for egg freezing and the related funding gaps [37–41], lay persons largely support the selective funding, viewing the funding of medical egg freezing more favorably than EEF subsidy [33, 37, 41–44]. As a result of this policy, some women cannot access EEF due to its high cost [42, 43, 45], rendering EEF part of stratified reproduction, available mostly to wealthier women in wealthier countries. Still, some scholars have expressed reservations towards EEF funding on grounds of competing health needs and the public message regarding the import of childbearing, in the light of resource scarcity that plagues public healthcare systems. The low usage rate of preserved eggs is another source of reluctance.

## Elective egg freezing in Israel

Marrying and having children are key to normative adulthood in Israel [46, 47]. In 2019, only 11% of women aged 45–49 never married [48]. Half the couples have children a year after they marry [48]. Single motherhood, mostly among women in their late 30s or early 40s, though relatively rare (7% of babies vs. 42% in the EU [49, 50];), also proliferates [51] as do same-sex families ([52], Triger 2016). Israel’s fertility rate is twice that of the OECD: 3.1 vs. 1.7 children per woman, respectively. Unparalleled funding policy of fertility treatment is another component of this distinct reproductive landscape. Israel offers nearly unlimited free IVF and ICSI^1^ treatments to any Israeli woman, up to the age of 45, until she has two children with her current partner, if applicable. Basic private insurances cover treatment towards the third and fourth children. Undergoing roughly five times more treatment cycles per capita than women in the EU and 12 times the U.S. average [53], Israeli women have been the world’s heaviest consumers of IVF for years. Given the high age limit for funded treatment, about a third of local IVF users (33–42%) are in their forties, having a slim chance of live birth [54]. This state of affairs has led some Israeli scholar to question whether this usage pattern, though equitable, is ethical [55, 56]. Israel’s pronatalism has often been attributed to the Biblical commandment “Be fruitful and multiply”, the Holocaust trauma and the perennial wars (e.g., [57]). Whatever the reason, the exceptional local fecundity and ART usage signify the intensity of the parental imperative.

In this local context, EEF flourishes. Israel’s Ministry of Health (MoH) ratified the application of egg freezing for non-medical reasons already in 2011, when EEF was still labelled experimental internationally [19]. The state regulations allow women aged 30 to 41 to undergo up to four treatment cycles, until they have preserved 20 eggs. The treatment is privately funded, with prices originally ranging between 3000USD and $6500 per cycle. In recent years, prices have declined, currently amounting to $1400–$5000 per cycle, visibly less expensive than in the U.S. (roughly $10,000 [19];), Canada [58], the UK [59] or the Netherlands [60]. Still, weighed against local women’s average salary of roughly $2500 in 2015 (mid-term of the price ranges cited above [61];), the cost is substantial, rendering service inequitable.

Beyond the financial burden, the lack of subsidy is symbolically significant. Given the similarity between EEF and IVF, the policy actually allocates funds by the immediate purpose of the treatment rather than the actual procedure that is being performed. Notably, when asked, Israeli students supported private funding of EEF, wishing to keep reproductive choice private, beyond state or employers’ reach [44].

Despite the private funding, EEF flourishes in Israel as well. Already in 2015, most local IVF units were performing EEF [62] and demand has been increasing consistently [63]. In the following, I track the construction of equity in the context of EEF in Israel’s distinct reproductive landscape.

## Method

The paper presents a discourse analysis by synthesizing three types of materials: 1. Press articles 2. A Parliamentary committee discussion and 3. Ethnographic interviews with Israeli women who have undertaken EEF. The specific sources are:

1. Press materials were selected as following:

The first three articles that topped google search of the terms: “Ynet egg freezing” in Hebrew, in December 15, 2021. Ynet is one of Israel’s most popular news and general content portal in the Hebrew language [64, 65]. The three top articles were (by order of Google list):

i. Dviri, M. You turned 35 and have not yet had egg freezing? You should read this, July 1, 2021.
ii. Passo, D. Why I decided to freeze eggs: 35 years Danielle Passo shares her fertility preservation, January 21, 2021.
iii. Kahane, A. Egg freezing: the methods that will help you stop the biological clock, September 15, 2020.

The second set of articles topped google search for “Haaretz, egg freezing.” Haaretz is Israel’s oldest daily newspaper, generally identified as highbrow and liberal, with 4–6% of local readership [66]. The three top Haaretz items were:

iv. Gal, S. Should you put all the eggs in one basket? January 31, 2021.
v. Mashiach, S. Anat Kamm, egg freezing is liberation from the clock tyranny, December 1, 2021.
vi. Efrati, I. The number of women undertaking egg freezing has grown tenfold in 6 years, February 6, 2018.

2. A discussion in the *Knesset* (Israeli Parliament) Committee for the Advancement of Women’s Status on EEF funding, held in November 30, 2021, lasting over 2 h. The meeting was fully videotaped and posted on the internet, with participants’ names and titles fully listed.
3. Interview material from a bi-national U.S.-Israeli qualitative study of women who have undertaken EEF or MEF, conducted between 2014 and 2016 [19]. In Israel, participants were recruited from three IVF clinics. The clinics’ staff phoned EEF patients, inviting them to participate in the study. Women who volunteered to take part were then contacted by phone by the author, who set a time and place for the interview at the women’s convenience. All interviews were conducted by the author and all participants signed written informed consent forms, agreeing to a confidential, audio-recorded interview in a private setting. Out of the nearly 200 women in the study, 36 Israeli participants have undertaken EEF. These interviews are being analyzed in the present article.

All three types of materials – articles, Parliamentary discussion and interviews – were analyzed with special focus on funding and related issues. Press articles were read and re-read and funding related sections were outlined. These sections were probed for emerging themes and a code was devised to capture these themes. The articles were then coded accordingly. Though various themes appeared in numerous items, each article’s framework was maintained and findings were presented chronologically per articles. The Parliamentary discussion, which was wholly devoted to the subject of EEF funding, was watched several times. The various arguments were identified and grouped according to their underlying rationale and the speaker’s role and position vis-à-vis EEF. The interviews were transcribed and a coding scheme was co-developed on the basis of emerging themes and patterns that were identified through careful review and comparison of all interview transcripts. Descriptive statistical analysis of the participants’ socio-demographic data was also compiled.

These materials neither exhaust nor represent Israel’s discourse on EEF and equity. In fact, they rather have several limitations: the press articles are few; the Parliamentary discussion is but a single event; the interviewed women are also few and have undertaken EEF several years ago, when the technology was newer and dearer. Moreover, all the interviewed women were Jewish. Though this religiously monolithic profile is typical of Israel’s EEF clientele, it is nonetheless incomplete. Still, taken together, the various sources reveal several themes of interest, underlying notions of equity, healthcare and reproduction in contemporary Israel.

## Findings

### The media

The more EEF proliferated in Israel, the greater the media attention it received. In Ynet, Israel’s popular news and general content website, the top-ranking article in a Google search conducted in 2022, was published in September 15, 2020, 6 months into the Covid pandemic and 3 days before the Jewish New Year, a highly sensitive moment for many single women in Israel ([47], 89). The article was authored by a local fertility expert, under the title: “Egg freezing: The methods that will help you stop the biological clock” [67]. The expert depicted the fertility decline following 35 and pointed at two possible fertility preservation responses: retrieve eggs and either have them fertilized or not before freezing. The gynecologist’s explanation assumed that founding a genetically related heteronormative family was women’s universal goal:

In case the woman meets a partner later in life, preserving unfertilized ova enables fertilization by the partner’s sperm, so the embryo carries the genetics of both parents... If the eggs were frozen after fertilization, no further procedure is needed before transfer to the womb. The disadvantage is that the embryo’s genetic makeup has been determined, even if the woman has found a new partner [67].

Cost and equity were not mentioned.

The second article was published 2 days later, a day before the Jewish New Year [68], depicting the author’s own encounter with EEF, as her friends encouraged her to undertake the procedure:

You’ll surely meet someone eventually and you’ll fall in love and get married, but meanwhile, you should do it, just to be on the safe side ... After all, you’ll soon turn 35.

The author decribed how she kept hearing about EEF

in the morning TV shows, in articles reviewing the EEF trend, in posts of celebrities who decided to freeze. Sometimes I feel that everyone is out there, queuing in front of the giant, scary freezer.

After many conversations, including with her mother, the author defied:

I am afraid, that’s the truth. I am afraid of being pregnant, let alone of having a baby come out of me, and I’m afraid that … I’ll be an anxious mother … Anyway, I don’t recall anyone pressuring Sarah, our Matriarch, to have egg freezing, and there she was, conceiving at 100. So for now, I can be left alone. Until then, I’ll go on being a terrific aunt, and rest assured that my nephews greatly enjoy my gifts, just saying.

Thus, despite the amplifying effect of the pandemic and the imminent High Holiday, the author publicized her decision to forego EEF. Still, she softened her rejection by attributing it to personal fears rather than a critical ideology. Moreover, she positioned herself within the Jewish collectivity by invoking “Sarah, our Matriarch” and eventually presented her motherly side by “being a terrific aunt.” The rejection of EEF was thus construed as a personal particularity rather than a critique of the technology or the policy. The article revolved around maternity. Cost and equity were not mentioned.

The third article, authored by a fertility clinician, overviewed the reasons to undertake EEF. It opened with a praising: “The State of Israel is an international fertility empire and a reproductive superpower” [69]. Yet, the doctor alerted that

A decade after the ratification of EEF, not all women of reproductive age are aware that the woman’s fertility potential is time restricted.

Taking for granted that motherhood is genetic and universally desired, the expert emphasized that women’s fertility was fast decaying. EEF cost or equity were not mentioned here, too.

*Haaretz*’s egg freezing articles were topped by a 2018 (Efrati) front page article, published under the title: “The number of women who have their eggs preserved has risen tenfold within six years”. Alongside the growing demand for EEF, the journalist cited women’s gratitude for the opportunity to “gain control over my life” and become mothers later on, when they will have met a partner. Experts were cited praising EEF as a solution for childless unpartnered women, who previously “had to choose between becoming a mother now or give up the motherhood dream.” A clinician decsribed births following EEF by women who had met a partner after performing the procedure, “showing that women who undertake EEF have not necessarily given up on couplehood.” A member of Knesseth who has undertaken EEF herself, enthusiastically called for funding, as did a practitioner, who described a “sweeping trend” towards EEF among religious women. The calls for funding did not mention equity.

The second article, published in January 31, 2021 [70], overviewed EEF. Clinicians criticized the policy’s age limit of 41 as unrealistic and condemned media misrepresentations of Hollywood stars’ late pregnancies, which omitted medical interventions and nurtured unrealistic optimism. Some women, all highly educated, acknowledged their wrongful relief after hearing of celebrities’ late pregnancies. Two sociologists pointed at the tacit enhancement of the bio-maternal imperative encapsulated in Israel’s ART policy and the encouragement of EEF. A researcher of Israel’s religious sectors warned that highlighting EEF

turns us into walking wombs and prompts questions. Does EEF eternalize gender inequality, as men have no such ‘expiry date’? will EEF become a prerequisite for dating? Or make women wait longer, for they have ‘an insurance’?

The issue of cost was mentioned twice. Two media celebrities, who had shared their EEF experiences online, highlighted EEF’s financial burden. Additionally, a professor of economics criticized “the state’s obsession with fertility, which allocates resources irrationally … We are one of the world’s densest countries and prosperity and welfare are injured by pronatalism. We should definitely not encourage natality!” At the same time, he accepted single women’s claim: “if the state is pronatalist, then it should go all the way. They are right, from their own perspective, but you don’t correct one distortion with another.” The journalist summarized by juxtaposing EEF’s benefits “for women in their early thirties who want to start a family somewhat later” alongside its clinical and financial challenges and invited debate and subtler policy making.

The third article in Google’s Haaretz’s list was a response to a previous one, published several hours earlier. The former article was authored by Anat Kamm [71], a woman famous for having leaked IDF documents several years earlier, for which she was sentenced to 4 years imprisonment. Kamm’s article, entitled: “Maayan Adam in the Service of Israel Egg Forces,” referred to a media journalist who had shared her EEF experience online and participated in the Knesseth Committee discussion on EEF funding, the day before. Kamm criticized Adam for having said, in the Knesset Committee discussion, that by undertaking EEF, women “will be able to sign for longer military service” and for defining EEF as feminism and a state interest:

The Israeli woman’s womb does not belong to her; it belongs to the state, as a greenhouse for future soldiers or an organ that needs subsidy to be set aside for several years, so its container, the woman, can serve the army for longer years … Why should we let, not to say invite the state into our womb?... the state can justly claim that if it funds the pregnancy, it controls it … left wing activists should cry out: ‘Get out of our womb, the state of Israel!’”

In a matter of hours, Haaretz published a response article, written by Israel’s IVF pioneer gynecologist [72], who dismissed Kamm’s arguments as “reflecting foundational and essential lack of understanding in reproductive medicine.” [73]. The clinician focused on equity:

women’s biological clock is cruel … especially after the age of 37 … the likelihood of conceiving plunges whereas the risk for congenital anomalies soars. EEF has profoundly transformed women’s options, for the better … enabling women to find a partner who is truly worthy of being their children’s father, without making painful compromises. It is unfair that the right to parenthood will be granted only to wealthy women … Moreover, women who had not had their eggs frozen, who are forced to undergo fertility treatments at relatively advanced ages, ‘cost’ the taxpayer way more, due to the enormous difficulty to conceive at an older age, when the eggs are fewer and of poor quality.

The practitioner thus portrayed women’s bodies as decaying, took for granted women’s universal quest for a genetically-related heteronormative family, and presented EEF as the cost-effective alternative to futile IVF cycles at a later age. Having established the import of EEF, he criticized the inequity of private funding. Notably, the speaker has been running one of Israel’s oldest and costliest private fertility clinics.

To sum up, in the popular *Ynet* portal, EEF was presented as a solution for childless women lacking a partner at reproductively advanced age. The articles centered on the very existence of the treatment and did not mention costs whatsoever. In the highbrow *Haaretz*, journalists assumed acquaintance with the treatment, discussing its advantages and challenges, including mixed views regarding the issues of cost and equity.

### State politics: the Knesseth committee discussion

Maayan Adam, a media celebrity who had shared her EEF experience online, approached a member of Knesset (MK) and proposed to discuss EEF funding. In November 30, 2021, the Parliament Committee for the Advancement of Women’s Status dedicated a meeting to the subject. The discussion chair was a female MK from the liberal Labor party. The participants included fertility experts, two women who had shared their EEF experiences online, a former MK who had undertaken EEF and had previously called for funding, representatives of Israel’s HMOs, a representative of an NGO for fertility treatments in Israel, the fertility preservation director of Puah (an Institute for reproduction in Jewish families^2^) and an MoH representative. The chairwoman opened the discussion by declaring her support of EEF subsidy, in the name of

the importance of state responsibility in this domain [reproduction]. Today’s discussion is convened in order to clarify the importance of subsidizing [EEF].

The participants raised various arguments in favor of subsidy: it would cohere with the state’s prioritization of natality, it would save the state “at least 4.8 million shekels ($1.5 million) a year” in unsuccessful IVF cycles to reproductively older women; EEF for women in their early 20s would reduce costs of genetic testing, as well. One clinician construed EEF as “preventive medicine, which is always better than curative treatment;” another one, as “a feminist issue today, like contraception in previous generations.” Maayan Adam, who had shared her EEF experience online, tied EEF to women’s confidence “to take a job, even in the army, to sign for longer years,” thereby reducing domestic economic violence, for women will no longer depend on their partners’ wealth.

Several participants invoked the issue of cost in conjunction with the state’s interest in fertility, which they interpreted as state responsibility to secure every woman’s ability to conceive. Other participants claimed that just as the state supports women needing other types of fertility treatments, it should assist single women who require EEF. The chairwoman placed equity center stage:

it is inconceivable that economic considerations will influence decisions regarding parenthood … the choice of parenthood cannot possibly depend on money!

The former MK who had undertaken EEF reiterated: “every woman has the right to decide about her future fertility irrespective of financial considerations.” A senior clinician followed suit: “it’s a distortion, that only women who can afford it, can undertake EEF”. The MoH representative reiterated: “we all want to prevent economic discrimination.” Notably, when the MoH representative mentioned that women postponed EEF to their later 30s, the chairwoman interrupted in protest:

but you are placing the issue again with the woman, while we are saying: ‘No! This is a state responsibility!’

Israel’s exceptional IVF funding was repeatedly mentioned as a reference point and a national accomplishment to be sustained. A consensus evolved that EEF should be partly subsidized, probably 1–2 cycles to women aged 34–36, thereby optimizing public expenditure and women’s reproductive outcome.

Eventually, towards the end of the discussion, the MoH officer demonstrated the state’s pronatalism by detailing the recent expansion of eligibility criteria for funded medical egg freezing, beyond cancer patients. At that point, however, she reported that the MoH Service Basket Committee ranked EEF at the lowest priority for funding,

which means that it is no longer being discussed … The Basket Committee saw the figures. They weighed and prioritized this funding request vis-à-vis others as they saw fit.

Thus, in practice and in contrast to the expressed support, the decision-making state body viewed EEF as a low priority service. The meeting has consistently sustained and ended with a consensus regarding the necessity and fairness of EEF funding that would increase local healthcare equity.

A Google search of the term ‘egg freezing and the name of the discussion’s chair (in Hebrew, August 26, 2022), was topped by a Ynet article entitled: “A [women’s magazine’s] journalist in a Parliament discussion: ‘Only a woman who has money can freeze eggs. This is a privileged procedure’” [74]. The subtitle described the “flood” of messages that the journalist had received “from women across all sectors who were unable to afford the costly procedure.” The second item was the Knesseth discussion’s minutes. The third was an article in a religious-nationalist newspaper, entitled: “A Parliament discussion on egg freezing: financial support will ease the loneliness” [75]. The titles left no doubt regarding the authors’ support of subsidy.

### Voices from the field: views of Israeli users of EEF

As elsewhere, nearly all the Israeli women who had undertaken EEF were well-educated, unpartnered and childless when they embarked on EEF in their later 30s [12]. Still, funding was a concern. Several participants have themselves raised the issues during the interviews. In the other interviews, the question was presented by the interviewer. Before turning to the women’s accounts, it is worth noting that over half the Israeli participants (19/36) had the procedure fully (17) or partly (2) funded by their parents.

Nearly all the Israeli participants who had undertaken EEF agreed that the procedure should be funded, at least in part. Sigal, 40, an educational counselor, found private funding unfair:

A country like ours, that believes in democracy and equality, should support women … if not full funding, at least some help. As my mother said: to have all the options open to **all** its citizens, so yes, the state should fund egg freezing!

The view of fertility as a state interest was repeatedly illustrated by its exceptional IVF funding, against which, the lack of subsidy for EEF became unacceptable. Gali’s approach was typical:

What annoyed me the most is that I had to pay, because I’m having precisely the same treatment like someone who’s undergoing insemination [=embryo transfer]! … If I’d fertilized my body, I wouldn’t have to pay! And the state encourages birth! I’m doing, like, egg freezing for my fertility, why should I pay the full cost?

Gilly, a school teacher, attributed the unfairness to the users not being “a couple, or a family by the book,” while Hagit, a system analyst, followed the same line and attributed EEF inequity as part of a wider discrimination:

single women are always discriminated against, in tax, in the way society looks at them. It’s hard enough as it is, and such funding could contribute to the equality.

Rose, a senior manager, politicized the inequity by contrasting it with the state’s generosity towards women in other life circumstances:

There are many women, well-educated women, who say: “I won’t have children until I can support them” … That’s why I’ve waited, built a career … and am now making a lot of money … But other women … cannot pay astronomical sums … And here stands an orthodox woman, who keeps bearing children … and she gets this whole treatment for fifty dollars, because for her it’s not EEF but IVF, and this does get funded! And this demoralizes the woman who works full time and encourages the one who doesn’t!!... It is **so** frustrating.

Debbie summed up: “I feel like a second-class citizen.”

Other women were, however, more ambivalent regarding state subsidy. Interestingly, these more critical women have not mentioned fairness or equity. Moriah, a graduate student, hesitated, out of concern that EEF funding might burden the state budget and compromise women’s reproductive autonomy. However, eventually, she thought that HMOs were making money out of childbearing and therefore should fund EEF. Adi, 36, an actress, called for a broader change. She, too, did not mention equity:

It all depends on education. The attitude towards fertility should change, there should be more choice, free choice. There’s too much intervention.

Yael, a project manager, saw EEF as a personal endeavor of mature women and as such, rightly self-funded:

See, at this age, you are already sort of ‘spoiled.’ So if you want to have egg freezing, you should pay for it.

Sharon, a high-tech lawyer, was reluctant as she did not think that EEF addressed a medical problem. Two other women, both newcomers to Israel, rooted their ambivalence in Israel’s wider reproductive scene. Emily, 35, from the UK, claimed that “the pressure here is very strong, so it’s very difficult to say ‘I don’t want children’, extremely difficult.” Jennifer, a doctor from the U.S., was the only one who questioned Israel’s IVF policy up front:

I’m not sure if the state should share [EEF] cost. There’s also a question about IVF and how much the state should fund... And there’s a broader question if one can be a parent at any age, things like that. Of course, when it comes to one’s private case, no one cares and just wants everything for oneself.

On the whole then, EEF users, who called for state subsidy, hailed reproduction as a state interest, as manifested in the exceptional IVF policy and therefore claimed that the state must ensure that every woman could conceive, irrespective of her economic and family circumstances. From this perspective, lack of funding was perceived as inequitable. In contradistinction, the few women who voiced reluctance about EEF subsidy, questioned existing policies and ideologies, including the maternal imperative, but have not invoked the issue of equity.

## Discussion

Starting with the more reluctant views, we note that speakers who disproved of EEF subsidy raised a range of arguments to ground their opinions, e.g., condemnation of the interference in women’s reproductive lives, critique of Israel’s ‘distorted’ IVF policy and “obsession with fertility,” enhancing the local bio-maternal imperative. At the center of the discourse, the speakers have placed broader issues related to the local reproductive policy and ideology. None of these speakers has referred to inequity as grounds for rejecting subsidy.

In contrast, advocates of state subsidy repeatedly brought up the issue of equity. Portraying Israel as a singular microcosm where childbearing is of exceptional import to individuals as well as the state, these actors endorsed inclusive language when claiming that the state was responsible of ensuring that local women of all material and family circumstances could access the treatment they needed in order to conceive. Subsidy supporters construed EEF funding as a civil issue and as such, deserving public funding in order to be equitable. The exceptional funding of IVF served as proof of the unfairness of denying state support from women who required EEF. Any link between a woman’s financial standing and maternal plans was repudiated as illegitimate. The fact that the majority of the potential users were singles – a life situation widely viewed in child-centered Israel [46], as elsewhere (e.g., [76]), as a lesser life situation [77] – further served the inequity claims. The issue at stake was universal motherhood. On the ground, the state has ruled out EEF subsidy, via the MoH Basket Committee.

A historical perspective might help elucidate the seeming contradiction vs. IVF policy. Israel’s IVF policy was formed in the 1980s, when, the country’s population was less than half its current size (4 vs. nearly 10 million [78];). Today, Israel is one of the world’s densest countries, expecting further massive growth in the near future, due to its elevated fertility and incoming migration. This demographic expansion is expected to result in major infrastructure challenges (e.g., [79]). Nonetheless, all attempts to modify the local IVF policy have failed. Already in 2003, Benjamin Netanyahu, then Treasury Minister, proposed to stop funded IVF for the second child but swiftly withdrew the proposal following public protest [80]. In 2005 and once again in 2013, experts have recommended rationing IVF, e.g., by limiting the number of cycles to women over 41 or lowering the age limit for funded treatment to 44. These suggestions and similar ones have also prompted forceful resentment that left the policy unchanged [81, 82]. Still, as recently as in 2020, a senior MoH officer admitted that “no Minister of Health wants to be associated with such a move” [81]. It may thus be suggested that the state’s interest in increasing fertility has probably receded along the decades. However, changing its IVF policy has proven too costly politically.

If this is indeed the case, then refraining from EEF subsidy, as decided by the MoH Basket Committee, may be highly adaptive. Economically, it saves state resources, at least in the short term; politically, EEF is not as entrenched in local expectations and its funding has never stirred a major public protest; symbolically, the denial of funding may gently signal a shift in the state’s demographic interest.

How should we read the fact that equity was mentioned exclusively by subsidy advocates? On its face, this is obvious: people who seek greater equity demand state support in order to secure equitable access to service. The case at hand is, however, atypical. EEF is a treatment serving young healthy women seeking to relieve a problem that is social rather than medical. The nature of the clientele was indeed visible on the public scene: the women who have undertaken EEF were journalists, celebrities and a former MK, alongside clinicians in lucrative practices. One might argue that this social profile reflects the private funding, which bars women of lesser means from the costly treatment.

Relevant statistics might shed some light. First, Israel’s poorest women concentrate in Arab and Jewish ultra-orthodox communities. Though more religious and even Ultra-orthodox Jewish women undertake EEF [83], women in these sectors are still less likely to undertake this treatment due to cultural-religious constraints. Moreover, women in these sectors marry almost invariably in their 20s. Second, women who more often remain single in their latter thirties in Israel are ones with higher education (with the exception of women with extremely low education who have not married young [84];, 49), whose income is generally, reasonably comfortable. Thus, Israeli women who might wish to undertake EEF are commonly highly educated, unpartnered, traditional or secular women in their later thirties and are relatively comfortable economically. The language of inclusiveness and the emphasis on equity and civil right, might therefore apply, primarily, to one, relatively privileged sector. Funding treatment for this particular sector might thus be considered as effectively expanding inequality rather than increasing equity by supporting women who are privileged to begin with.

The economic aspect of this generalization might be challenged by recalling that in the aforementioned bi-national U.S.-Israel study, over half the Israeli participants had their EEF funded by their parents. This finding might be reconciled with the current argument in several ways. First, cost of living in Israel is extremely high and even well-educated single women who work full time may find it hard to save for exceptional expenses like EEF. Second, judging by the interviews, parental funding did not necessarily represent the woman’s financial limitations. In some cases, it was rather a symbolic statement of parents’ support of their daughter’s decision. Third, several women were actually unsure whether they wanted to have children and pursued EEF largely because of their parents’ requests. Fourth, some women maintained very close ties with their parents, rendering economic boundaries somewhat blurred. Either way, the parents’ funding also attests to the relative strength of the women’s families of origin. Indeed, some of the women in the study were working in less remunerative jobs as they somewhat relied on their parents’ wealth. Beyond these explanations lies the decrease in EEF cost, which has roughly halved since the time of the bi-national study, rendering the procedure affordable to a wider clientele. Within this perspective, a state subsidy might be seen as directed to serve a relatively well-educated well-earning segment of the population.

To further contextualize the subsidy demand, one should consider the wider context of Israel’s healthcare. Though offering full national coverage, the service is far from equitable. Some sectors have been experiencing unequal access and care for years [85]. Infant mortality, for instance, is nearly thrice a prevalent in the south of Israel as compared with its center (5.3 vs. 1.9 death per thousand live births respectively) and life expectancy is roughly 7 years shorter in poorer communities (83 vs. 75.7 years [86];). Hospital care is suboptimal and varies greatly [87], in fact, inequity has been pointed at as one of the system’s gravest problems [85, 87]. With the demographic growth and increasing privatization, health gaps and inequity further expand [88, 89]. During the Covid crisis, some of these inequities have surfaced poignantly (e.g., [90, 91]). As for the material conditions of children in Israel: nearly 30% of Israeli children live below the poverty line. In the city of Jerusalem, the respective figure soars to 53%. Over a fifth (21.1%) of local children suffer food insecurity [92].

Against this backdrop, calling for EEF subsidy in the name of equity raises several questions: How pressing is EEF vis-à-vis other local health needs and resources? How difficult is it for potential local users to fund EEF on their own, now that prices have dropped? What message would EEF subsidy convey to Israeli citizens regarding the state’s view of the acuteness of childbearing? These questions would stand out especially sharply given the lack of subsidy for EEF in public healthcare systems elsewhere, including in countries much wealthier than Israel (with the noted recent exception of France). Indeed, IVF is funded in Israel more generously than elsewhere. Yet, IVF funding dates back to the early 1980s, it aims to relieve a medical problem and is more efficient in terms of actual natality. All these aspects will gain further significance with the current challenges brought about by the formidable demographic growth.

And yet, one can well understand the perspective of Israeli women who call for EEF subsidy. Studies have shown that women who undertake EEF in settings where IVF is reimbursed, feel discriminated against by what they view as policy inconsistency [37, 40]. This is especially striking in the case at hand. Israeli women who seek EEF have seen many of their peers partner and eventually undergo IVF, practically free of charge. However, when they themselves, unpartnered, wished to preserve their fertility potential, they had to pay considerable amounts for the treatment. Against the routinized free IVF, EEF fee may reasonably be experienced, as some women have described, as a toll on unpartnered women at a reproductively advanced age. From a broader perspective, these single women face intense pressures, probably stronger than those encountered by their counterparts in other OECD countries. On the one hand, they need to make a living in an extremely costly environment. As of 2021, real purchasing power of Israeli wages was 20% below the OECD average and 44% lower than in the U.S. Prices in Israel were 17 and 40% higher than in the U.S. and the Euro zone, respectively [93]. Women, who earn, on average, 32% less than local men [94], grapple with especially severe material challenges. At the same time, Israeli women are subject to explicit and implicit pressures to become mothers and sustain various sorts of criticism and sanctions if they do not abide by this local imperative [46]. Within this setting, the call for funding would seem but a quest to align EEF policy with that of IVF, so as to grant single Israeli women equitable reproductive rights.

Is there a more general lesson to be learned about equity from the highly particular case of EEF in Israel? The bi-national egg freezing study may be instructive. When invited to recommend improvements to treatment, U.S. study participants suggested a whole range of complementary services, like stand-alone EEF clinics, after working-hours information sessions and injection classes, diverse staff specializing in EEF for single, lesbian, ethnic, and religious minority women, transportation and home care on day of egg retrieval, in-house psychologists specializing in EEF needs of single women, especially ones left by their partners. Alongside, insurance coverage and fee reduction were also mentioned. Israeli participants, in contrast, have concentrated almost exclusively on the need for public subsidy [19].

The sharp difference outlines each group’s distinct perception of ideal healthcare. In the U.S., where healthcare is primarily private and commercial, the participants have listed a range of supportive services, aimed at improving women’s treatment experiences. The Israeli women, in contrast, have all been used to public healthcare, which, despite considerable erosion, forms the backbone of local health services. U.S. participants have thus basically accepted EEF as a privately funded service but called for an improved treatment envelope. Israeli women have not even envisioned such services, but were appalled that they had to pay. The women’s expectations thus reflected the systems they have been using, with American participants expecting more comprehensive and supportive service than Israelis, who had lower expectations but protested against inequitable access.

Equity thus emerges in its context embeddedness. People’s views of what is fair and equitable are cast by their experience-based perceptions. What is considered inequitable and raises public resentment in one context might not be considered an option at all in another. The very use of the concept of ‘equity’ as a strategic campaigning tool may therefore teach us about local systems’ scope and priorities. “Health equity – underpinned by social justice – needs to be embedded at the structural level to support effective prioritization,” contended van Roode et al. [95]. Yet, ‘social justice’ and ‘effective prioritization’ are context-specific terms that require local definitions and grounding. Thus, in Israel, universal access to healthcare, including reproductive treatments, if at a basic standard, is so deep-seated that numerous users, activists, media persons, religious authorities and even policy makers supported state subsidy for EEF, a treatment that addresses a social rather than a medical condition, encountered primarily by a relatively well-established subpopulation.

Future studies may assess the impact of existing EEF funding policy on local women and on local notions of health and equity. The answers may inspire a wider investigation of the health policy-equity nexus and its sensitivity as reflecting and influencing local notions underlying healthcare funding.

## Conclusion

The present study reveals the crucial weight of local contexts in the shaping of perceptions of desirable healthcare and subsequently, of notions of health equity. Thus, in one context, private funding of a specific service may be taken for granted as ‘natural’ while in another context, paying for the same service out of one’s own pocket – even a much lower sum – may create a sense of discrimination among users and invoke calls for equity in the name of fairness and social justice. As such, the circumstances in which calls for equity are invoked may serve as a sensitive tool for learning about local ideals of healthcare and about public expectations from local services, as well as the diversity of notions of state responsibility, fairness and equity. At times, state actual responses to such calls for equity and the rift between these responses and public declarations may also point at emerging, possibly not-yet-declared changes in state policies and interests.

## Data Availability

Media coverage and full recording of the analyzed Parliamentary Committee meeting are publicly available on the internet. All reference details are included in the manuscript. Interview transcripts are kept confidentially in the author’s files and are available from the author, subject to confidentiality limitations.

## Footnotes

1 Physical insertion of a sperm into a retrieved egg.

2 The organization is “devoted to helping the greater Jewish community fulfil their dreams of building a healthy family.” https://www.puahfertility.org/about#story.
